# Enantioseparation and molecular docking study of selected chiral pharmaceuticals on a commercialized phenylcarbamate-β-cyclodextrin column using polar organic mode

**DOI:** 10.1038/s41598-023-41941-5

**Published:** 2023-09-07

**Authors:** Máté Dobó, Márk Ádám, Béla Fiser, Lajos Attila Papp, Gergely Dombi, Khaled Sekkoum, Zoltán-István Szabó, Gergő Tóth

**Affiliations:** 1 Department of Pharmaceutical Chemistry, Semmelweis University, Hőgyes E. u. 9, 1092 Budapest, Hungary; 2 Higher Education and Industrial Cooperation Centre, University of Miskolc, Egyetemváros, 3515 Miskolc, Hungary; 3 Ferenc Rakoczi II Transcarpathian Hungarian College of Higher Education, Beregszász, Transcarpathia Ukraine; 4 Department of Physical Chemistry, Faculty of Chemistry, University of Lodz, 90-149 Łódź, Poland; 5 Department of Pharmaceutical and Therapeutic Chemistry, George Emil Palade University of Medicine, Pharmacy, Science and Technology of Târgu Mures, Târgu Mureş, Romania; 6 Bioactive Molecules and Chiral Separation Laboratory, Faculty of Exacte Sciences, University Tahri Mohamed of Bechar, PO Box 417, 08000 Bechar, Algeria; 7 Department of Drugs Industry and Pharmaceutical Management, George Emil Palade University of Medicine, Pharmacy, Science and Technology of Târgu Mures, Târgu Mureş, Romania; 8 Sz-Imfidum Ltd., 525401 Lunga, Romania

**Keywords:** Analytical chemistry, Characterization and analytical techniques, Molecular capsules

## Abstract

The chiral separation capability of Chiral-CD-Ph column, containing phenylcarbamate-β-cyclodextrin as the chiral selector in polar organic mode was investigated. A total of twenty-five compounds with different structures and acid–base properties were evaluated, and twenty of them were separated using acetonitrile or methanol as eluent. The effects of various chromatographic parameters, such as the type and proportion of organic modifier, flow rate, and column temperature were analyzed in detail in relation to chromatographic performance. A U-shape retention curve was observed when a mixture of acetonitrile and methanol was used as the eluent, indicating different types of interactions in different solvent mixtures. Van 't Hoff analysis was used for calculation of thermodynamic parameters which revealed that the enantioseparation is mainly enthalpy controlled; however, entropic control was also observed. The enantiomer recognition ability at the atomic level was also investigated through a molecular docking study, which revealed surface binding in polar organic mode instead of inclusion complexation. Our work proves that the phenylcarbamate-β-cyclodextrin-based chiral stationary phase can be effectively used in polar organic mode for the chiral separation of structurally diverse compounds. Furthermore, it is important to note that our study demonstrated that surface binding is responsible for the formation of supramolecular complexes in certain cyclodextrin derivatives.

## Introduction

The surge in the number of enantiopure drug approvals has led to the increasing regulatory requirements. In such cases, it is crucial to provide high enantiomeric purity to ensure their safety and efficacy. Trace amounts of the inactive enantiomer, or the distomer, may also be present as impurities^1,2^. Chiral separation is also important in the food and fragrance industries, where enantiomers of certain compounds can have varying aromas or flavors. Separation techniques, such as gas chromatography, high-performance liquid chromatography (HPLC), supercritical fluid chromatography, and capillary electrophoresis are widely used for enantioselective analysis. Among them, HPLC with chiral stationary phases (CSPs) is considered the gold standard in the field^3–5^.

Cyclodextrins (CDs) are among one of the most widely used chiral selectors. CDs are cyclic oligosaccharides formed by d-glucopyranosyl units linked through 1,4-linkages. These macrocycles feature a toroidal shape with a hydrophobic interior cavity and hydrophilic exterior. Due to their chiral nature, they are capable of enantiodiscrimination through transient diastereomeric complex formation either through inclusion complexation or surface binding^6^. In liquid chromatographic enantioseparations, the choice of mobile phase can impact the binding mode to the chiral selector. In an aqueous system, inclusion complexation is more likely. The hydrophobic cavity of the CD provides a confined environment for the lipophilic part of the guest molecule. This inclusion effect shields the guest molecule from the surrounding aqueous environment, leading to a decrease in its entropy. When CD-based CSPs are used in polar organic or normal-phase modes, the inner cavity is obstructed by solvent molecules, preventing inclusion complexation. Nevertheless, in such media, hydrophilic interactions may be enhanced, where solutes with hydrophilic groups bind to the polar surface of the CD. Nevertheless, without the application of additional techniques such as molecular modeling or spectroscopy, determining the binding mode is not possible^5^. Diverse types of CDs are available, including native- and derivatized CDs. The latter were developed to offer additional intermolecular interactions, such as π–π interactions, hydrogen bonding, dipole–dipole interactions, and ion-pairing, resulting in improved enantioseparation capabilities^7,8^. Although there is a continuous interest in the development and implementation of new CD derivatives as silica bound CSPs, it is worth noting that only a limited number of these are commercially available^9^. Armstrong et al. made significant contributions to the field by developing both native and derivatized α-, β-, and γ-CD CSPs, which enabled the efficient enantioseparation of dansylated amino acids, barbiturates, as well as aromatic and aliphatic sulfoxides through high-performance liquid chromatography (HPLC)^10–12^. After Armstrong's research group demonstrated that CD-based stationary phases are suitable for the direct separation of enantiomers, numerous new developments were initiated. Wang et al. successfully developed native β-CD, permethylated β-CD, and perphenylcarbamated β-CD CSPs for the enantioseparation of several dansyl amino acids, flavonoids, β-blockers, and α-ionone derivatives^13^. Additionally, Tang's group has studied the enantiomeric separation of various analytes including flavonoids, aromatic alcohols, β-blockers, and amino acids using three β-CD CSPs prepared by clicking different per-chloro-methyl-phenylcarbamated moiety onto a silica support^14,15^. In a recent article, Li et al. prepared a novel 3,5-dichloro-phenylcarbamate-β-CD chemically bonded CSP and used it for the enantioseparation of different proton-pump inhibitors using normal-phase LC^9^. With the help of the 3,5-dimethylphenylcarbamate-β-CD column, Rezenka et al. were able to determine the presence of two diacylglycerol enantiomers in bacteria, suggesting the existence of heterochiral membranes^16^. At present native CD-based columns as well as acetylated β-, permethylated β-, dimethylated β- hydroxypropylated β-, napthylethylcarbamated β-, 3,5-dimethylphenylcarbamate-β-, and perphenylcarbamate-β-CD are commercially available on the market as CSPs.

The Chiral CD-Ph column features a phenylcarbamated β-CD-modified silica CSP. It is produced by Osaka Soda (Shiseido) and distributed by Phenomenex. The column has already been used in several cases for the separation of different enantiomers for a specific purpose, such as besifloxacin hydrochloride^17^, carvone^18^, citalopram^19^, fexofenadine^20^, omeprazole and 5-hydroxyomeprazole^21^. However, a comprehensive study investigating the utilization of this commercialized column for diverse components has not yet been published. Further clarification is needed regarding the performance characteristics of the column.

One of the key advantages of using CD-based CSPs in chiral separations is the ability to utilize multimodal elution, which enables the resolution of a wide range of stereoisomeric compounds through the combination of different elution modes, including reversed-phase, normal-phase, and polar organic-mode^22^. Several studies recommend using reversed-phase or normal-phase mode with CD-based CSPs^23,24^. However, phenylcarbamated CDs, similar to polysaccharide selectors with phenylcarbamate groups, can also exhibit good performance in polar organic mode^25–27^. Despite this, inferior performance was reported with in-house prepared phenylcarbamated-β-CD column in polar organic mode according to previous literature^28^. In polar organic mode, the mobile phase consists solely of polar organic solvents, such as pure alcohols (methanol (MeOH), ethanol (EtOH), and 2-propanol (IPA)) or pure acetonitrile (ACN), individually or in combination. To prevent the formation of ionic species, an acid (e.g., acetic acid (Acac)) or a base (e.g., diethylamine (DEA)) can be introduced to the mobile phase, depending on the analyte's acid–base properties. Polar organic mode offers several advantages, including shorter run times, high efficiency, easier mass spectrometric coupling, and enhanced solubility of the analytes in the mobile phase. Additionally, polar organic mode is suitable for both analytical and preparative applications^29–33^. The present study aimed to investigate the enantioseparation performance of the Chiral CD-Ph column in polar organic mode for a range of structurally diverse compounds and to clarify the mechanism of chiral recognition through a molecular docking study.

## Materials and methods

### Materials

**Figure 1:**
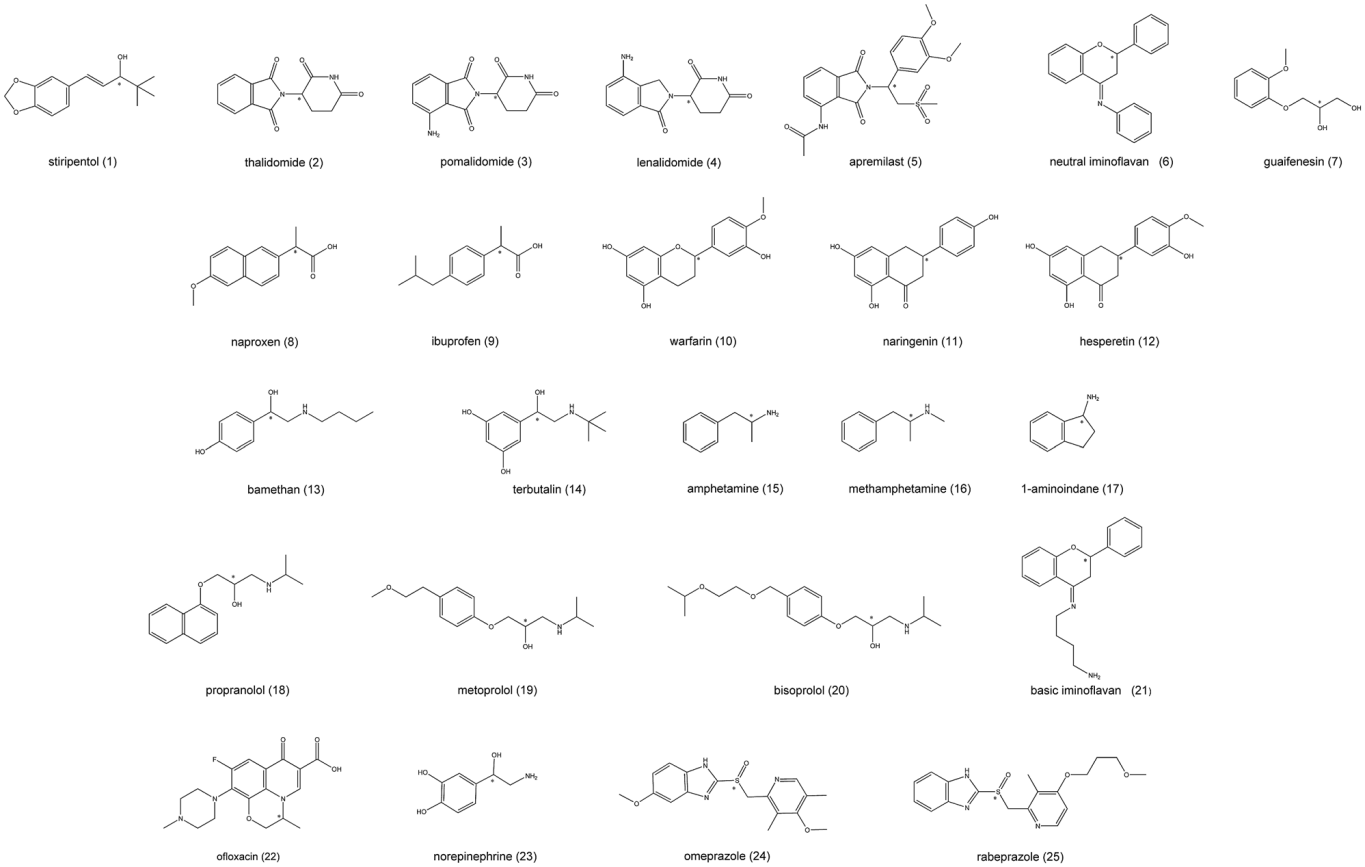
Chemical structures of the studied test compounds (chiral centers marked by an asterisk).

Racemic thalidomide, pomalidomide, (*S*)-pomalidomide, (*R*)-rabeprazole, lenalidomide, (*R*)- and (*S*)-apremilast were purchased from Beijing Mesochem Technology Co. Ltd. (Beijing, China). (*R*)-thalidomide, (*S*)*-*enantiomer-enriched lenalidomide^34^, guaifenesin, warfarin, ibuprofen, 1-aminoindane, amphetamine sulfate, methamphetamine hydrochloride, bamethan hemisulfate, terbutaline sulfate, propranolol hydrochloride, metoprolol tartrate, norepinephrine hydrochloride, and omeprazole were ordered from Sigma-Aldrich, Hungary (Budapest, Hungary). Stiripentol, naproxen, naringenin, hesperetin, bisoprolol hemifumarate, ofloxacin was from TCI Chemicals (Tokyo, Japan). Rabeprazole sodium was United States Pharmacopoeia (USP) reference standard (Rockville, USA). Iminoflavan derivatives were synthesized as previously described^35,36^. The structure of investigated compounds is depicted in Fig. 1, while certain physico-chemical parameters are summarized in Table 1. Gradient grade MeOH and ACN as well as Acac and DEA were purchased from Thomasker Finechemicals Ltd. (Budapest, Hungary). Chiral CD-Ph column (250 × 4.6 mm, particle size 5 μm), Nucleodex β-PM column (200 × 4 mm, particle size 5 μm), and Lux amylose-1 column (150 × 4 mm, particle size 5 μm) were ordered from Phenomenex (Torrance, CA, USA). The Astec CYCLOBOND I 2000 Chiral HPLC Column (250 × 4.6 mm, particle size 5 μm) was purchased from Sigma-Aldrich, Hungary.

**Table 1:** Calculated physico-chemical parameters of the studied compounds.

No.	Compound	log*P*	p*K*_a_	Acid–base character
1	Stiripentol	2.94	–	Neutral
2	Thalidomide	0.33	–	Neutral
3	Pomalidomide	0.02	1.6	Neutral (weak base)
4	Lenalidomide	− 0.4	2.3	Neutral (weak base)
5	Apremilast	1.86	–	Neutral
6	Neutral iminoflavan	5.54	–	Neutral
7	Guaifenesin	1.39	–	Neutral
8	Naproxen	3.18	4.2	Acidic
9	Ibuprofen	3.97	5.3	Acidic
10	Warfarin	2.70	5.0	Acidic
11	Naringenin	2.52	7.9	Neutral (weak acid)
12	Hesperetin	2.60	7.9	Neutral (weak acid)
13	Bamethan	1.09	10.0	Basic
14	Terbutaline	0.90	9.8	Basic
15	Amphetamine	1.76	9.9	Basic
16	Methamphetamine	2.07	9.9	Basic
17	1-aminoindane	1.61	9.2	Basic
18	Propranolol	3.48	9.4	Basic
19	Metoprolol	2.15	9.7	Basic
20	Bisoprolol	2.20	9.5	Basic
21	Basic iminoflavan	4.41	9.7	Basic
22	Ofloxacin	− 0.39	6.7 (basic); 5.4 (acidic)	Amphoteric
23	Norepinephrine	− 1.24	8.9 (basic) 9.5 (acidic)	Amphoteric
24	Omeprazole	2.23	9.4 (acidic); 4.8 (basic)	Amphoteric
25	Rabeprazole	0.6	9.4 (acidic); 4.2 (basic)	Amphoteric

### LC-UV analysis

LC-UV analysis was conducted on two different HPLC system. A Jasco HPLC system consisting of PU-2089 plus quaternary pump, AS-4050 autosampler, MD-2010 diode array detector, Jetstream 2 Plus thermostat with JASCO ChromNAV software and an Agilent 1260 Infinity HPLC system consisting of G1312B binary gradient pump, G1367E autosampler, G1315C diode array detector with MassHunter B.04.00 software. During the screening phase, a uniform flow rate of 0.5 mL/min and a column temperature of 20 °C was used. All stock solutions were prepared at 1 mg mL^−1^ in the mobile phase (MeOH or ACN), and further dilutions were made using the same solvent. An injection volume of 1 μL was used for each measurement, with three parallel measurements performed in each case. We performed the detection at the UV absorption maximum of each material. The enantiomer elution order (EEO) was determined by injecting enantiomers of known absolute configuration. It should be noted that not all compounds had an enantiomerically pure form available to us.

The retention factor (*k*) was determined as k = (t_R_ − t_0_*)*/t_0_, where *t*_R_ is the retention time for the eluted enantiomer, and *t*_0_ is the dead time. Selectivity (α) was calculated as k_2_/k_1_. The resolution (R_s_) was calculated with the following formula: R_s_ = 2(t_2_ − t_1_)/(w_1_ + w_2_), where t_1_ and t_2_ are the retention times, w_1_ and w_2_ are the extrapolated peak widths at the baseline. The columns' hold-up time (t_0_) was determined using 0.1% Acac (v/v) dissolved in MeOH and detected at wavelengths of 210 or 256 nm.

### Molecular docking study

**Figure 2:**
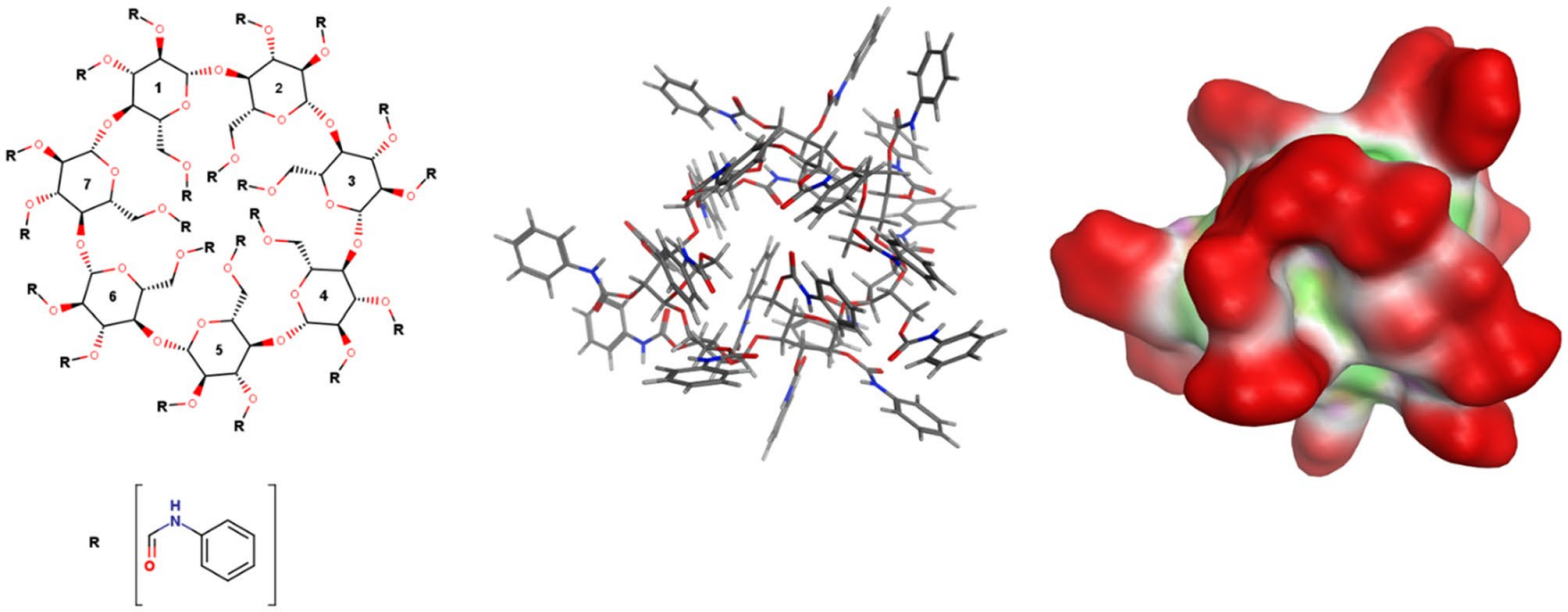
2D (left) and 3D (middle) representations of phenylcarbamate-β-cyclodextrin. R = phenylcarbamate. The molecular surface of the 3D structure (right) is also presented, where red indicates exposed regions (to the solvent), green signifies hydrophobic groups, and magenta represents polar groups.

The perphenylcarbamoylated-β-CD (phenylcarbamate-β-CD chiral selector) (Fig. 2) was optimized by energy minimization applying the OPLS-AA force field^37^ along with the Born implicit solvation model. MarvinSketch was used for drawing 2D representation of the studied host (Marvin version 21.15.7, MarvinSketch Iodine 7, ChemAxon, https://www.chemaxon.com).

The optimized phenylcarbamate-β-CD structure has been used as a host in docking calculations. The 3D structures of thalidomide, pomalidomide, lenalidomide, stiripentol, hesperetin, metoprolol, norepinephrine, and rabeprazole enantiomers were prepared and the corresponding PDBQT files were created which includes information on the torsional degree of freedom, atomic partial charges and atom types, polar hydrogens, but not hydrogens bonded to carbon atoms. These files were used as inputs docked to the prepared phenylcarbamate-β-CD by using AutoDock Vina^38^ and the binding affinity of the complexes was computed. A maximum of 9 binding modes were considered for each enantiomer and the exhaustiveness of the calculations was set to 8. The best binding modes (lowest in terms of energy) were selected, and the corresponding binding affinities (*E*_A_) were compared. The grid box was 30 × 30 × 30 Å^3^ in each case.

## Results and discussion

### Screening in polar organic mode

**Figure 3:**
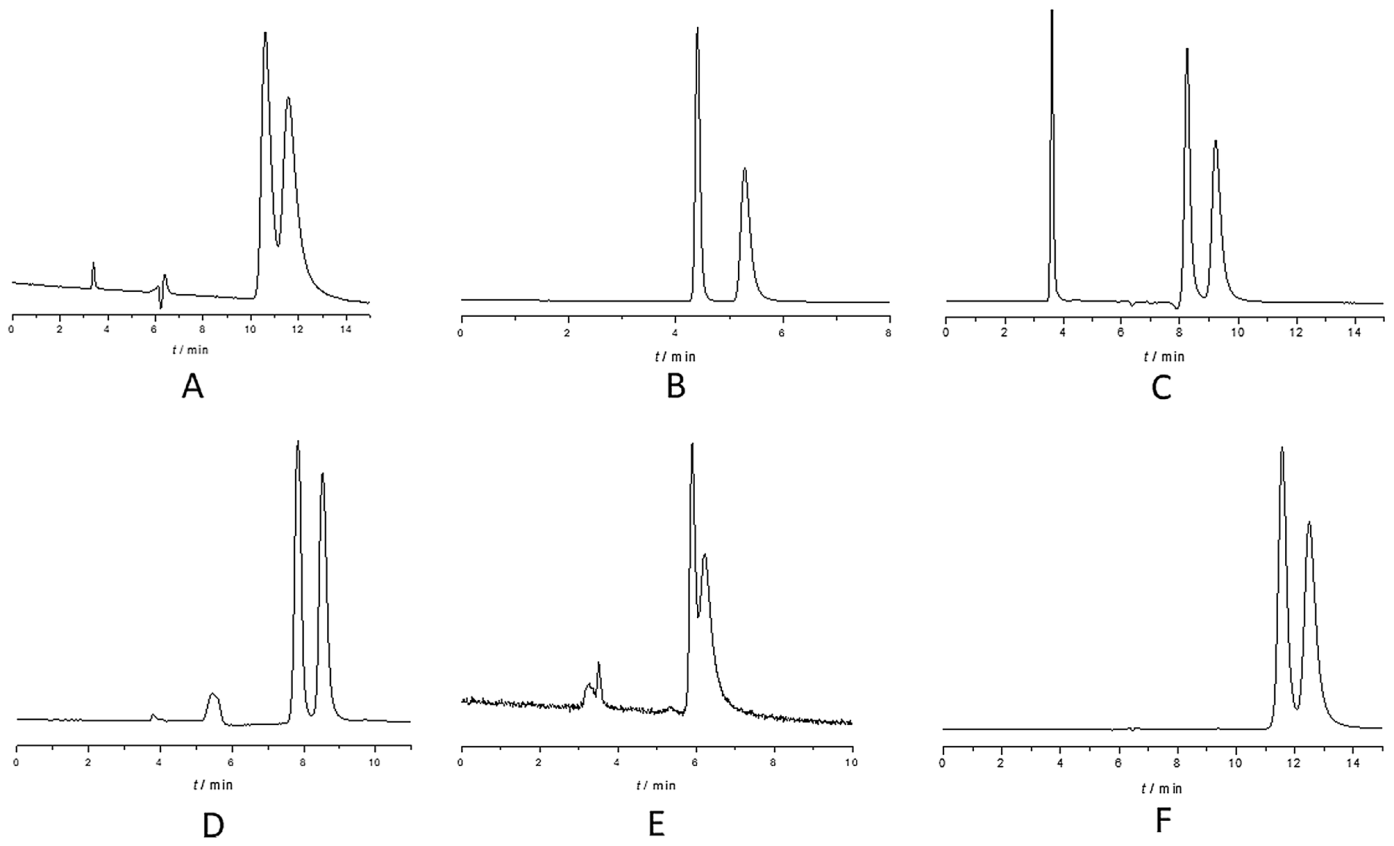
Representative chromatograms on Chiral CD-Ph column using constant 0.5 mL/min flow rate and 20 °C. (**A**) lenalidomide in MeOH, (**B**) stiripentol in MeOH, (**C**) bisoprolol in MeOH with 0.1% DEA (v/v), (**D**) basic iminoflavan in MeOH with 0.1% DEA (v/v), (**E**) terbutaline in MeOH with 0.1% DEA (v/v), (**F**) Rabeprazole in MeOH with 0.1% DEA (v/v) and 0.1% Acac (v/v).

To evaluate the performance of the Chiral CD Ph column in polar organic mode, 25 compounds were selected with diverse chemical structures and acid–base properties. ACN or MeOH was used as eluent, supplemented with 0.1% Acac (v/v) for acidic compounds and 0.1% DEA (v/v) for basic compounds. For amphoteric compounds, both modifiers (Acac and DEA) were used in equal proportions (0.1–0.1% (v/v)). The obtained results are summarized in Table 2, and representative chromatograms are presented in Fig. 3.

**Table 2:** Chromatographic data during the screening method on Chiral CD-Ph column (k_1_—retention factor of the first eluting enantiomer, k_2_—retention factor of the second eluting enantiomer, α—selectivity, R_s_—resolution).

	MeOH*				ACN*			
	k_1_	k_2_	α	R_s_	k_1_	k_2_	α	R_s_
Stiripentol	0.17	0.41	2.41	3.7	0.28	0.47	1.68	1.7
Thalidomide	2.95	–	–	–	1.20	1.51	1.26	0.7
Pomalidomide	2.36	–	–	–	1.29	1.76	1.36	1.1
Lenalidomide	1.61	1.86	1.16	1.1	3.36	3.90	1.16	0.3
Apremilast	3.23	–	–	–	0.41	–	–	–
Neutral iminoflavan	2.07	2.80	1.35	5.8	1.30	1.37	1.05	0.9
Guaifenesin	2.28	3.57	1.57	4.5	0.16	0.27	1.69	1.7
Naproxen	1.96	–	–	–	1.62	–	–	–
Ibuprofen	0.45	–	–	–	1.37	–	–	–
Warfarin	0.25	0.28	1.12	0.5	0.51	–	–	–
Naringenin	0.93	1.00	1.07	0.6	0.71	–	–	–
Hesperetin	1.41	2.04	1.45	3.0	1.25	–	–	–
Bamethan	1.69	2.00	1.18	1.6	1.15	1.46	1.27	1.3
Terbutaline	0.80	0.88	1.10	0.7	0.34	–	–	–
Amphetamine	2.05	2.85	1.39	1.7	2.13	2.21	1.04	0.4
Methamphetamine	0.91	1.13	1.24	1.5	1.41	1.50	1.06	0.3
1-Aminoindane	1.05	1.15	1.10	0.6	0.20	0.24	1.20	0.3
Propranolol	1.18	–	–	–	0.94	–	–	–
Metoprolol	1.16	1.44	1.24	1.6	1.60	1.72	1.08	0.4
Bisoprolol	0.91	1.26	1.38	2.1	1.89	2.32	1.23	0.6
Basic Iminoflavan	1.16	1.62	1.40	4.6	0.92	1.00	1.09	0.9
Ofloxacin	3.89	–	–	–	1.11	–	–	–
Norepinephrine	0.29	0.53	1.83	1.5	0.11	–	–	–
Omeprazole	0.60	0.65	1.08	0.4	0.25	0.31	1.24	0.5
Rabeprazole	0.61	0.74	1.21	1.5	0.20	–	–	–

*For neutral compounds (**1**–**7**) pure eluent, for acidic compounds (**8**–**12**) the eluent was modified with 0.1% Acac (v/v), for basic compounds (**13**–**21**) the eluent was modified with 0.1% DEA (v/v), for amphoteric compounds (**22**–**25**) the eluent was modified with the mixture of DEA-Acac 0.1–0.1% (v/v).

The results in Table 2 show that the Chiral CD-Ph column exhibits enantiorecognition towards 20 out of 25 racemic compounds tested. Notably, twelve compounds were separated with baseline resolution (*R*_s_ ≥ 1.5) without requiring any method development. For five compounds, including iminoflavan derivatives, hesperetin, guaifenesin, and stiripentol, the resolution exceeded three. However, for five compounds, including thalidomide, warfarin, naringenin, 1-aminoindane, and omeprazole, the obtained enantioresolution values were lower than 0.7.

To ensure comparability with other commercially available columns, we repeated the same measurements on three additional chiral columns. Among these, two are CD-based CSPs, commercially available in the market (Astec Cyclobond I 2000, containing β-CD as chiral selector, and Nucleodex β-PM, containing permethylated β-CD as chiral selector). Lux Amylose-1 was the third column, a polysaccharide-type CSP, containing amylose tris(3,5-dimethylphenyl)-carbamate as chiral selector. The latter being generally recognized as one of the most effective chiral columns available on the market. Enantioseparation results obtained on these three columns are summarized in Supplementary Tables 1, 2, and 3, respectively. The results clearly demonstrate that in polar organic mode, the performance of the Chiral CD-Ph column is significantly superior compared to other CD-based CSPs. Using the Astec Cyclobond I 2000 and the Nucleodex β-PM columns, only a total of nine enantioselective separations were observed, but these were generally characterized by poor resolution values. The highest enantioresolution value achieved on these columns in polar organic mode was a mere 1.2 (warfarin on Astec Cyclobond I 2000, using ACN-based mobile phase). The obtained results indicate that CD-based columns, where the structure of the chiral selector contains no aromatic groups, exhibit subpar performance in polar organic mode. Among these columns, ACN tends to yield better results than MeOH due to the absence of aromatic rings that could potentially form π–π interactions in alcohol-based mobile phases. In contrast, not surprisingly, out of all four columns, the Lux Amylose-1 demonstrated the most remarkable performance, frequently yielding very high resolution values. Nonetheless, the performance of the Lux Amylose-1 column and the Chiral CD-pH column is comparable. A comparison of enantioseparation systems using three different characterization approaches is illustrated in Supplementary Fig. 1. Our results clearly indicate that the Chiral CD-Ph column is a promising choice for enantiomer separation in polar organic mode.

It is also noteworthy that three out of the five compounds where enantioseparation was not observed, all present a carboxylic group in their structure (naproxen, ibuprofen, and ofloxacin). This observation is interesting and may suggest a correlation between the presence of a carboxylic group and the lack of enantiorecognition on the Chiral CD-Ph column in polar organic mode. Upon comparing the performances of the separation systems using ACN and MeOH-based eluents, it can be easily observed that MeOH appears to be more effective than ACN for these analytes, particularly for acidic compounds. However, there are some analytes (thalidomide, pomalidomide, omeprazole) that show better results with ACN-based mobile phases. Thus, it is recommended to investigate both eluents for screening. Based on the results from the sample set employed, no clear pattern can be discerned regarding retention factors or enantioselectivity. From the data, in most cases, the retention factor is not lower in ACN than in MeOH. Additionally, we can find examples where a low retention factor is paired with high enantioresolution value (e.g., stiripentol), while in several cases, despite the high retention factor, enantioseparation was not observed (e.g., apremilast, omeprazole).

The compounds selected exhibit structural diversity, but certain groups of compounds have similar structures, such as thalidomide analogs (thalidomide, pomalidomide, lenalidomide, apremilast), flavanones (naringenin and hesperetin), β-blockers (propranolol, bisoprolol, metoprolol), proton-pump inhibitors (omeprazole and rabeprazole), iminoflavan derivatives, and the amphetamine-methamphetamine pair. The chromatographic behavior was compared within these groups. It was observed that the chromatographic behavior of amphetamine and methamphetamine, as well as the iminoflavan derivatives, is similar despite the differences in acid–base properties of iminoflavans. Thalidomide and pomalidomide exhibited related results, both compounds being separated only in ACN. However, lenalidomide, which has a minor structural difference of an oxo group compared to pomalidomide, also displayed enantioseparation in MeOH. In contrast, apremilast, which has significant structural variations from its parent compound, was not successfully separated. Naringenin and hesperetin exhibited similar chromatographic behavior and could only be separated using a MeOH-based eluent. However, while the two compounds are structurally similar, naringenin had a relatively low resolution of 0.6 compared to the notably higher resolution of 3.0 for hesperetin. Among the proton pump inhibitors, rabeprazole exhibits a higher resolution when separated in methanolic eluent compared to omeprazole. It is also worth noting that while omeprazole can be separated in ACN, rabeprazole cannot.

In conclusion, it can be inferred that predicting enantiomer separation on the Chiral-CD-Ph column is challenging as even slight structural modifications in the analytes can significantly affect the chromatographic outcome. However, this is also generally true for other chiral columns. Exploring the interactions between the selector and the selectand can aid in more accurately predicting the success of chiral separations^39,40^.

The influence of various chromatographic parameters, including eluent composition, eluent modifier, temperature, and flow rate on the enantioseparation performance was also investigated on the Chiral CD-Ph column, using a restricted set of analytes.

### The impact of various chromatographic parameters on chiral separation

For a more in-depth analysis, we have chosen to investigate a range of compounds including thalidomide derivatives and stiripentol as representative of neutral compounds, naringenin and hesperetin from acidic compounds, bisoprolol, metoprolol, and basic iminoflavan form basic compounds, and norepinephrine and rabeprazole from amphoteric compounds.

The effects of various eluent additives were thoroughly investigated, including the influence of MeOH–ACN mixtures on enantioseparation. Additionally, we evaluated the impact of changing flow rate and temperature on chromatographic performance. To study the impact of acidic additive, different percentages of Acac (0.05%, 0.1%, 0.15% (v/v)) were added to MeOH, and for the basic additive different percentages of DEA (0.05%, 0.1%, 0.15% (v/v)) were utilized. For neutral and amphoteric compounds, the effects of both modifiers were examined separately and in combination. In the case of neutral compounds, the addition of modifiers did not significantly influence the separation performance. However, for basic compounds such as bisoprolol and metoprolol, enantioseparation was only achieved in the presence of a basic modifier with the best resolution observed using 0.15% DEA (v/v). Furthermore, the basic iminoflavan compound was found to be baseline separated even in neat MeOH, which can possibly be attributed to its highly lipophilic nature and the large distance between the basic nitrogen and the chiral center (Supplementary Table 4). The addition of Acac in the case of naringenin and hesperetin improved resolution up to 0.1% Acac (v/v) (Supplementary Table 5). Our investigation of the amphoteric compound rabeprazole, revealed that enantioseparation is possible in pure MeOH, while this was not the case for norepinephrine. When using DEA as a modifier in MeOH, norepinephrine enantiomers were baseline separated, however, upon increasing the DEA concentration of the mobile phase, enantioresolution significantly worsened. Using Acac as modifier alone resulted in the lack of noticeable enantioseparation for amphoteric compounds. The best resolution was obtained using a combination of both 0.1% Acac (v/v) and 0.1% DEA (v/v) (Supplementary Table 6).

**Figure 4:**
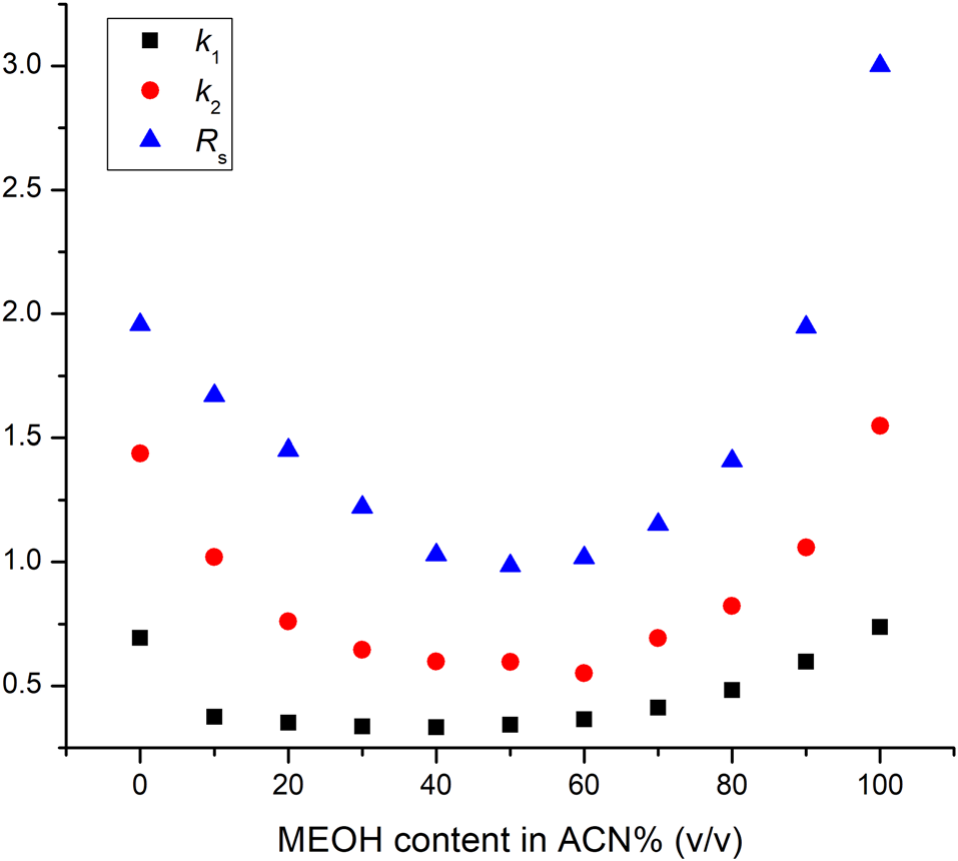
Plots of the retention and resolution factors as a function of the MeOH content on Chiral CD-Ph column. (Chromatographic conditions: flow rate, 0.5 mL/min; column temperature, 20 °C).

The impact of binary mobile phases containing varying percentages of MeOH and ACN on resolution and retention factors was also evaluated. Figure 4 shows the effect of MeOH content in ACN on the retention and resolution of stiripentol as an example (similar U-shaped retention curves were also observed for the other set of investigated analytes). It is interesting to observe that the lowest retention and resolution values were observed at 50% ACN content in MeOH (v/v). From this local minimum increasing the MeOH or ACN content led to an increase in enantioresolution. The highest resolution can be observed by applying 100% ACN or 100% MeOH. This U-shape curve indicates that the mechanism of separation is different in MeOH compared to ACN. This is explained by the fact that the probability of H-bond formation between the stiripentol enantiomers and the chiral selector is higher in ACN than in MeOH, where most probably π–π interactions are dominant. Similar observations have already been made on polysaccharide- and cyclofructan-based columns in polar organic mode^26,41,42^. Additionally, it should be noted that the polarity and the dielectric constant of the two solvents also play a role in determining the strength of the interactions between the enantiomers and the chiral selector, which can further influence separation performance.

Variations in flow rate did not elicit any unexpected results. As the flow rate increased, retention decreased while the selectivity remained unchanged. In general, a higher flow rate resulted in a slight reduction in resolution, but the retention times can be reduced, and the peak shapes can be improved by applying higher flow rates (Supplementary Fig. 2).

#### Thermodynamic analyses

The separation temperature is a critical factor that can significantly impact the selectivity, resolution, retention and EEO of enantiomers during HPLC enantioseparation. Thermodynamic calculations are highly significant and commonly employed methods for establishing a chiral recognition mechanism using direct HPLC. The aim of the thermodynamic study in our research was to provide insights into the interaction energies and driving forces behind chiral recognition on the Chiral CD-Ph column in polar organic mode. By employing thermodynamic calculations, we aimed to elucidate the underlying mechanisms that contribute to the enantioseparation efficiency observed in our experimental results^5,43,44^.

To investigate the energetic interactions between the analytes and the stationary phase a classical van 't Hoff approach was used, assuming only enantioselective interaction sites on the stationary phase. Van 't Hoff plots were constructed by plotting the natural logarithm of the retention factor as a function of the inverse of the absolute temperature over a temperature range of 10–40 °C (applying 10 °C, 20 °C, 25 °C, 30 °C, and 40 °C).

1 $$lnk=\frac{\Delta H^\circ }{RT}+\frac{\Delta S}{R}+ln\Phi$$

where *R* stands for the universal gas constant, *T* is the temperature in Kelvin, *k* is the retention factor of the enantiomers. Δ*H*° denotes the standard enthalpy, while Δ*S*° is the standard entropy change of transfer of the solute from the mobile phase to the stationary phase, and $\Phi$ is the phase ratio of the Chiral CD-Ph column. If Δ*H*° is constant in the selected temperature range, a linear relationship is obtained between ln*k* and 1/T, with a slope of − Δ*H*°/R and an intercept of Δ*S*°/R + ln $\Phi$. Since the value of the phase ratio is seldom known, Δ*S*°* values (Δ*S*°* = Δ*S*° + Rln $\Phi$) are often used, to compensate the uncertainty in $\Phi$^5,45^.

Similarly, the difference in change of standard enthalpy Δ(ΔH°) and standard entropy Δ(ΔS°) for the two enantiomers moving from the mobile phase to the stationary phase were also calculated according to modified van ’t Hoff equation:

2 $$ln\alpha =-\frac{\Delta (\Delta H^\circ )}{RT}+\frac{\Delta (\Delta S^\circ )}{R}$$

Isoenantioselective temperatures (T_iso_) were also calculated as the ratio between ΔΔH° and ΔΔS°. T_iso_ represents the temperature at which the enthalpy contribution is compensated by the entropic term, indicating that Gibbs free energy (ΔΔG°) equals zero. At T_iso_, the two enantiomers co-elute, and no separation is achieved. ΔΔG° provides information on the strength of binding between selector and selectant, more negative values indicating a more efficient binding. Consequently, a value of Δ(ΔG°) = 0 means that there is no difference between the binding strength of the enantiomers, therefore, at T_iso_ the two enantiomers co-elute, and no separation is achieved.

3 $$T\text{iso }= \frac{\Delta (\Delta H^\circ )}{\Delta (\Delta S^\circ )}$$

4 $$\Delta (\Delta G^\circ )= \Delta (\Delta H^\circ )-T*\Delta (\Delta S^\circ )$$

The obtained thermodynamic data are summarized in Table 3. By analyzing the obtained data, it is possible to gain valuable insights into the factors influencing enantioselectivity on the Chiral CD-Ph column and to optimize the separation conditions for enhanced selectivity and resolution.

**Table 3:** Calculated thermodynamic parameters on Chiral CD-Ph column (mean ± SD).

Compounds (eluent)	Equation	r^2^	Δ(Δ)H° (kJ/mol)	Δ(Δ)S° (J/mol K)	Δ(Δ)G° (kJ/mol)	T_iso_ (°C)	Q*
Thalidomide (ACN)	ln*k*_1_ = 717.79x − 2.062	0.9993	− 5.97 ± 0.08	− 17.15 ± 0.21	− 0.86 ± 0.02		
	ln*k*_2_ = 957.88x − 2.449	0.9995	− 7.96 ± 0.09	− 20.36 ± 0.27	− 1.89 ± 0.03		
	lnα = 240.04x − 0.386	0.9989	− 2.00 ± 0.10	− 3.21 ± 0.32	− 1.04 ± 0.04	348 ± 18	2.09 ± 0.07
Pomalidomide (ACN)	ln*k*_1_ = 656.23x − 1.797	0.9995	− 5.45 ± 0.12	− 14.94 ± 0.12	− 1.00 ± 0.05		
	ln*k*_2_ = 972.9x − 2.414	0.9992	− 8.09 ± 0.11	− 20.07 ± 0.09	− 2.10 ± 0.08		
	lnα = 316.66x − 0.617	0.9990	− 2.63 ± 0.15	− 5.13 ± 0.13	− 1.10 ± 0.12	240 ± 12	1.72 ± 0.05
Lenalidomide (ACN)	ln*k*_1_ = 852.43x − 1.660	0.9994	− 7.08 ± 0.03	− 13.76 ± 0.09	− 2.99 ± 0.09		
	ln*k*_2_ = 937.48x − 1.837	0.9999	− 7.79 ± 0.02	− 15.28 ± 0.08	− 3.24 ± 0.12		
	lnα = 85.05x − 0.182	0.9986	− 0.71 ± 0.03	− 1.51 ± 0.09	− 0.26 ± 0.08	195 ± 9	1.57 ± 0.02
Lenalidomide (MeOH)	ln*k*_1_ = 959.67x − 2.630	0.9989	− 7.98 ± 0.05	− 21.87 ± 0.14	− 1.46 ± 0.01		
	ln*k*_2_ = 1047.2x − 2.803	0.9992	− 8.70 ± 0.02	− 23.31 ± 0.11	− 1.76 ± 0.01		
	lnα = 87.538x − 0.1732	0.9929	− 0.73 ± 0.10	− 1.44 ± 0.07	− 0.30 ± 0.02	232 ± 17	1.70 ± 0.07
Stiripentol (MeOH)	ln*k*_1_ = 322.26x − 0.632	0.9994	− 2.68 ± 0.01	− 5.25 ± 0.09	− 1.11 ± 0.03		
	ln*k*_2_ = 935.17x − 2.391	0.9999	− 7.75 ± 0.03	− 19.87 ± 0.13	− 1.85 ± 0.08		
	lnα = 612.91x − 1.759	0.9933	− 5.09 ± 0.05	− 14.63 ± 0.18	− 0.74 ± 0.07	75 ± 4	1.17 ± 0.03
Stiripentol (ACN)	ln*k*_1_ = 624.16x − 2.266	0.9954	− 5.19 ± 0.02	− 18.84 ± 0.09	0.42 ± 0.08		
	ln*k*_2_ = 3329.8x − 10.216	0.9997	− 27.68 ± 0.19	− 84.94 ± 0.25	− 2.37 ± 0.09		
	lnα = 2705.6x − 7.949	0.9987	− 22.49 ± 0.25	− 66.10 ± 0.31	− 2.73 ± 0.12	67 ± 3	1.19 ± 0.03
Hesperetin (MeOH + 0.1% Acac)	ln*k*_1_ = 1318.9x − 4.093	0.9992	− 10.97 ± 0.11	− 34.03 ± 0.31	− 0.82 ± 0.07		
	ln*k*_2_ = 1781.7x − 5.257	0.9996	− 14.81 ± 0.09	− 43.71 ± 0.12	− 1.79 ± 0.08		
	lnα = 462.8x − 1.164	0.9988	− 3.85 ± 0.03	− 9.68 ± 0.51	− 0.96 ± 0.21	124 ± 12	1.31 ± 0.03
Naringenin (MeOH + 0.1% Acac)	ln*k*_1_ = 1127.6x − 3.8788	0.9995	− 9.37 ± 0.05	− 32.25 ± 0.21	0.23 ± 0.04		
	ln*k*_2_ = 1007.7x − 3.4024	0.9994	− 8.37 ± 0.05	− 28.29 ± 0.18	0.05 ± 0.01		
	lnα = − 119.95x + 0.4765	0.9990	0.99 ± 0.10	3.96 ± 0.44	− 0.18 ± 0.03	− 21.3 ± 4	0.84 ± 0.08
Metoprolol (MeOH + 0.1% DEA)	ln*k*_1_ = 1172x − 2.982	0.9990	− 9.74 ± 0.03	− 24.79 ± 0.05	− 2.35 ± 0.12		
	ln*k*_2_ = 937.33x − 2.305	0.9989	− 7.79 ± 0.04	− 19.16 ± 0.03	− 2.08 ± 0.10		
	lnα = 235.05x − 0.6278	0.9988	− 1.95 ± 0.07	− 5.22 ± 0.09	− 0.40 ± 0.10	101 ± 5	1.26 ±
Bisoprolol (MeOH + 0.1% DEA)	ln*k*_1_ = 803.93x − 2.1416	0.9990	− 7.45 ± 0.02	− 22.61 ± 0.12	− 0.70 ± 0.12		
	ln*k*_2_ = 1520x − 4.3218	0.9995	− 13.78 ± 0.03	− 41.85 ± 0.25	− 1.32 ± 0.20		
	lnα = 716.11x − 2.1802	0.9945	− 6.33 ± 0.05	− 18.13 ± 0.21	− 0.94 ± 0.05	76 ± 1	1.17 ± 0.01
Basic iminoflavan (MeOH + 0.1% DEA)	ln*k*_1_ = 1093.7x − 2.992	0.9991	− 9.09 ± 0.07	− 24.88 ± 0.05	− 1.68 ± 0.10		
	ln*k*_2_ = 1671.1x − 4.671	0.9992	− 13.89 ± 0.12	− 38.83 ± 0.09	− 2.32 ± 0.11		
	lnα = 577.39x − 1.679	0.9987	− 4.80 ± 0.18	− 13.96 ± 0.12	− 0.64 ± 0.15	71 ± 3	1.15 ± 0.02
Norepinephrine (MeOH + 0.1% DEA + 0.1%Acac)	ln*k*_1_ = 962.88x − 2.815	0.9995	− 8.01 ± 0.06	− 23.40 ± 0.19	− 1.03 ± 0.02		
	ln*k*_2_ = 1601.5x − 4.489	0.9997	− 13.31 ± 0.21	− 37.33 ± 0.02	− 2.19 ± 0.07		
	lnα = 638.58x − 1.675	0.9989	− 5.31 ± 0.27	− 13.93 ± 0.15	− 1.16 ± 0.18	108 ± 8	1.28 ± 0.03
Rabeprazole (MeOH + 0.1%DEA + 0.1%Acac)	ln*k*_1_ = 1311.8x − 3.629	0.9992	− 10.90 ± 0.12	− 30.17 ± 0.09	− 1.91 ± 0.09		
	ln*k*_2_ = 1427.5x − 3.915	0.9993	− 11.86 ± 0.17	− 32.55 ± 0.08	− 2.17 ± 0.08		
	lnα = 115.8x − 0.2856	0.9991	− 0.96 ± 0.13	− 2.37 ± 0.09	− 0.25 ± 0.15	132 ±	1.36 ±

*Q = Δ(ΔH°)/(T* Δ(Δ)S°)_298 K_.

**Figure 5:**
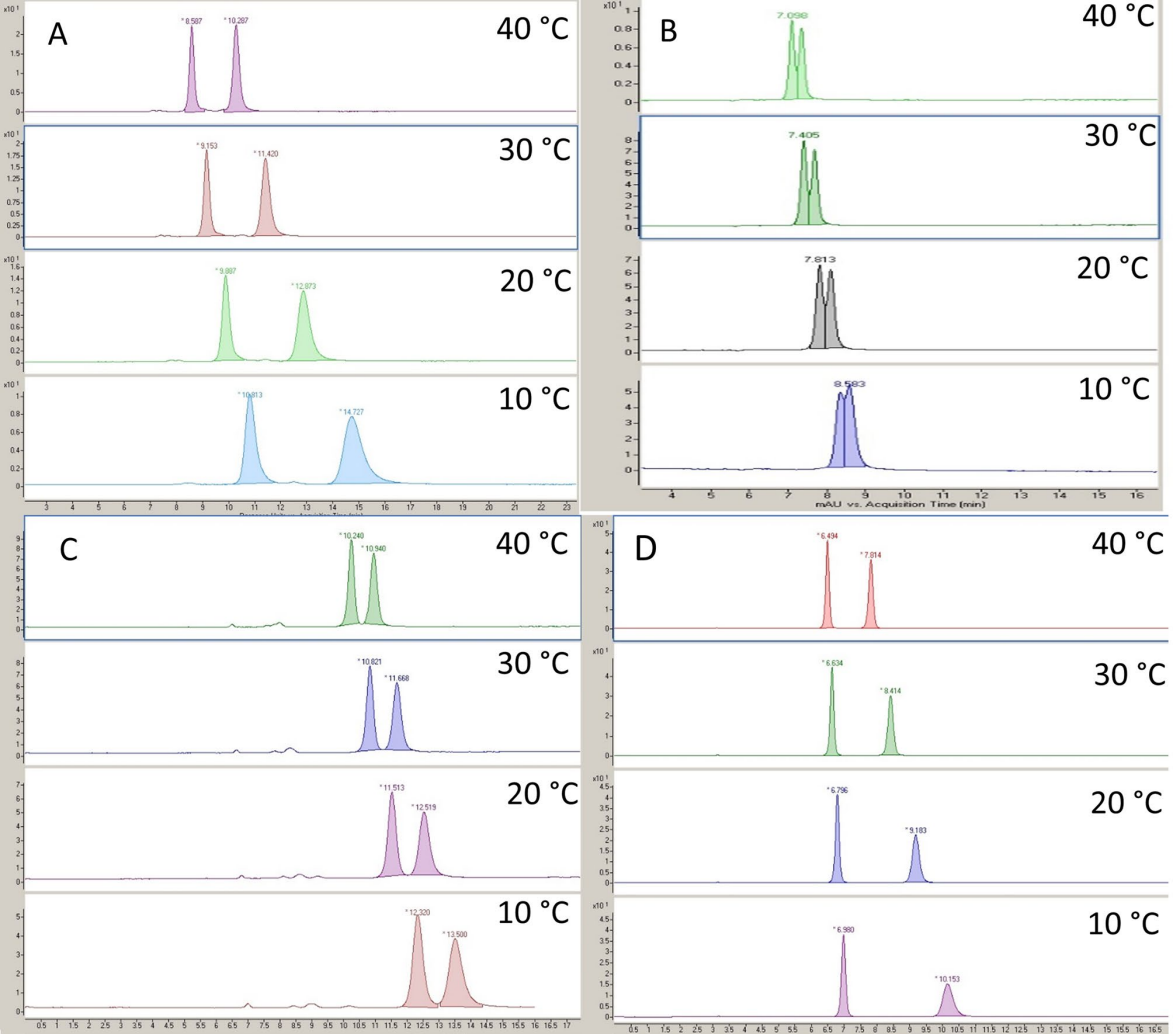
Representative chromatograms obtained from a thermodynamic study on the Chiral CD-Ph column at a flow rate of 0.5 mL/min. (**A**) Hesperetin with MeOH + 0.1% Acac; (**B**) Naringenin with MeOH + 0.1% Acac; (**C**) Lenalidomide with MeOH; (**D**) Stiripentol with MeOH.

Based on the data obtained at various temperatures, it is evident that the retention factor consistently decreases as separation temperature increases, irrespective of the analyte under examination. As expected, selectivity decreased with increasing temperature except for naringenin in MeOH-based mobile phase where selectivity slightly increased with increasing temperature. Representative chromatograms for the thermodynamic study are depicted in Fig. 5.

Linear regression analyses were performed for all cases, comparing ln*k* vs. 1/*T* and ln*α* vs. 1/*T*, indicating that the binding mode and conformation of the selector remained unchanged within the temperature range investigated. Supplementary Fig. 3 displays fitted van 't Hoff plots, illustrating the relationship between lnα and 1/T. The Δ(ΔH°) values provide insights into the relative ease of analyte transfer from the mobile phase to the stationary phase. These values ranged from − 22.49 kJ/mol (stiripentol in ACN) to 0.99 kJ/mol (naringenin in MeOH). The significantly low Δ(Δ*H*°) value observed for stiripentol in ACN suggests that the second eluted enantiomer forms an additional strong secondary interaction, such as a hydrogen bond with the selector, indicating relatively strong levels of chiral recognition. Negative Δ(ΔH°) values for enantiomeric pairs are accompanied by negative Δ(ΔS°) values. A negative Δ(ΔS°) reflects an increase in order and/or a reduction in degrees of freedom during enantioselective interactions between the selector and selectand. Just as the highest negative Δ(ΔH°) value is found for stiripentol in ACN, the highest Δ(ΔS°) value is also found for stiripentol in ACN, which is − 66.10 J/molK. The relative contributions of enthalpic and entropic terms to the free energy of adsorption can be elucidated by examining the enthalpy/entropy ratio, denoted as Q. By comparing the Q values, it was observed that the enantioseparation was predominantly controlled by enthalpy in most cases, as indicated by Q values larger than 1. Interestingly, for naringenin, enantioseparation controlled by entropy (Q < 1) were observed. This finding suggests that the binding of naringenin to the chiral selector may exhibit distinct characteristics compared to other analytes, potentially involving different interactions or mechanisms. Temperature-dependent EEO reversal was not observed. When the column temperature was T_iso_, the isomers would be eluted simultaneously. However, if the column temperature was higher than T_iso_, the EEO would be reversed. In all investigated cases the T_iso_ value is out of the range of use.

### Molecular docking study

**Figure 6:**
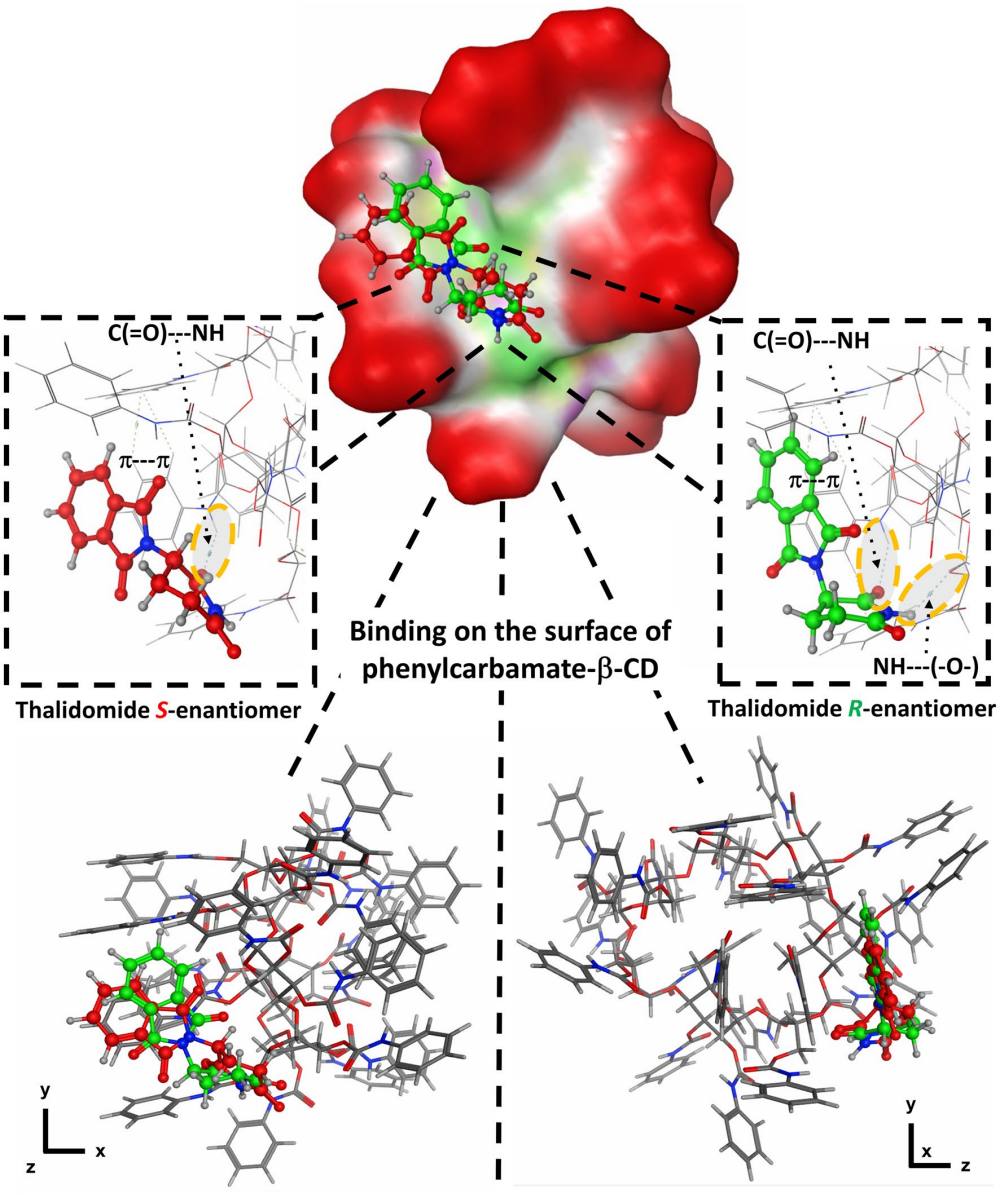
Interactions between the best binding modes of thalidomide enantiomers and phenylcarbamate-β-CD. The molecular surface of the 3D structure (right) is also presented, where red indicates exposed regions (to the solvent), green signifies hydrophobic groups, and magenta represents polar groups.

Molecular docking was performed to gain insight into the possible mechanisms of enantioseparation and chiral recognition on the Chiral CD-Ph column. The interactions between the enantiomers of eight analytes (thalidomide, pomalidomide, lenalidomide, stiripentol, hesperetin, metoprolol, norepinephrine, and rabeprazole) and phenylcarbamate-β-CD were investigated (Fig. 6).

The results indicate that the calculated binding energies are within the range of − 4.5 to − 7.2 kcal/mol. The negative values suggest that the formation of the host–guest complexes is a spontaneous process driven by enthalpy. It is important to emphasize that in all cases where the enantiomer elution order was determined, the molecular docking results and experimental findings are in good agreement with each other (Table 4). By analyzing the optimized structure of phenylcarbamate-β-CD it was found that the cavity of the CD host is blocked by the phenylcarbamate groups. Thus, surface binding occurred, and the analytes established interactions with the surface of the phenylcarbamate-β-CD (Fig. 6). For the clear presentation of the accessibility of the cavity in native CDs and phenylcarbamate-β-CD, the molecular surface of the 3D structure was depicted in Supplementary Fig. 4. Based on our this, attempting to “force” the analyte into the cyclodextrin cavity in case of the phenylcarbamoylated-CDs is an erroneous approach.

**Table 4:** Binding affinities (*E*_A_) of the *R* and *S* enantiomers of the studied analytes towards phenylcarbamate-β-CD. The types of main interactions are also listed along with the observed experimental enantiomer elution order where it is available.

	*E*_A_ (kcal/mol)		Observed enantiomer elution order
	(*R*)	(*S*)	
Thalidomide	− 7.1	− 6.6	S < R
Pomalidomide	− 7.2	− 6.7	S < R
Lenalidomide	− 6.7	− 6.3	S < R
Stiripentol	− 5.5	− 5.4	n.d.
Hesperetin	− 5.9	− 6.6	n.d.
Metoprolol	− 5.1	− 5.0	n.d.
Norepinephrine	− 4.7	− 4.5	n.d.
Rabeprazole	− 5.9	− 6.0	R < S

*n.d.* no data.

The difference in binding energy (Δ*E*_A_) between a pair of enantiomers can be considered a measure of chiral recognition, and a non-zero Δ*E*_A_ is a criterion for enantiomer separation. Although Δ*E*_A_ in some cases is only 0.1 kcal/mol (e.g. rabeprazole), the two enantiomers are still baseline separated. In theory, the Δ*E*_A_ values should correspond with the selectivity magnitudes obtained from the enantioseparation conditions, but this is not always the case. These discrepancies arise from the limitations of docking which prevents taking into account all experimental factors such as the impact of additives, solvation effects, unreacted silica particles in the columns, and others. Despite the listed limitations, docking calculations were able to predict the observed enantiomer elution order in four cases. Additionally, four additional analytes were also studied, but for those, experimental data regarding the enantiomer elution order were not available due to the absence of enantiomerically pure standards. Various types of interactions were established between the analytes and the host including stronger hydrogen bonds and hydrophobic contacts, and π–π interactions (Fig. 6). To validate the docking results, single-point energy calculations were conducted on specific complexes (such as pomalidomide–Chiral CD-Ph). The calculations utilized the APFD hybrid density functional, and the influence of solvent effects was considered by applying the conductor-like polarizable continuum model (CPCM) The outcomes revealed that the enantiomer energy hierarchy remained unchanged; however, there was an observed increase in relative energy (see Supplementary Table 7).

## Conclusions

It was found that the Chiral CD-Ph column demonstrated good performance in separating a diverse range of 25 racemic compounds with varying chemical structures and acid–base properties in polar organic mode. The high success rate in separating the tested compounds highlights the effectiveness of the Chiral CD-Ph column in polar organic mode, especially when using MeOH as the mobile phase. We investigated the influence of mobile phase compositions, additives, flow rate, and temperature on enantioseparation in detail and found that the appropriate mobile phase is crucial for optimal results. Based on our results, MeOH-based mobile phases lead to better outcomes than ACN-based ones. Thermodynamic analysis revealed mainly enthalpy-controlled enantioseparations. We also investigated enantiorecognition at the molecular level using molecular docking and found that molecules bind to the surface of the CD. Although there were some limitations to our study, such as the lack of enantiomerically pure standards for some analytes, docking calculations were still able to predict the observed EEO in several cases. Overall, our work demonstrates that the Chiral CD-Ph column is a reliable and versatile tool for chiral analysis in polar organic mode.

### Supplementary Information

Supplementary Information.

## Data Availability

The datasets used and/or analyzed during the current study available from the corresponding author on reasonable request.
