# Supplementary material for: Enantioseparation and molecular docking study of selected chiral pharmaceuticals on a commercialized phenylcarbamate-β-cyclodextrin column using polar organic mode
Source: Sci Rep. 2023 Sep 7;13:14778. doi: 10.1038/s41598-023-41941-5 (PMC10485059; doi:10.1038/s41598-023-41941-5)
Supplement: Supplementary file 1 — Supplementary Information. [file 41598_2023_41941_MOESM1_ESM.docx]

**SUPPLEMENTARY MATERIALS**

**Enantioseparation and molecular docking study of selected chiral pharmaceuticals on a commercialized phenylcarbamate-β-cyclodextrin column using polar organic mode**

Máté Dobó^1^, Márk Ádám^1^, Béla Fiser^2,3,4^, Lajos Attila Papp^5^, Gergely Dombi^1^, Khaled Sekkoum^6^, Zoltán-István Szabó^7,8^, Gergő Tóth*^1^

^1^ Department of Pharmaceutical Chemistry, Semmelweis University, Budapest, Hungary

^2^ Higher Education and Industrial Cooperation Centre, University of Miskolc, Egyetemváros, H-3515 Miskolc, Hungary

^3^ Ferenc Rákóczi II. Transcarpathian Hungarian Institute, Beregszász, Transcarpathia, Ukraine

^4^ Department of Physical Chemistry, Faculty of Chemistry, University of Lodz, 90-149 Łódź, Poland

^5^ Department of Pharmaceutical and Therapeutic Chemistry, George Emil Palade University of Medicine, Pharmacy, Science and Technology of Târgu Mures, Romania

^6^ Bioactive Molecules and Chiral Separation Laboratory, Faculty of Exacte Sciences, University Tahri Mohamed of Bechar, POBox 417, Bechar 08000, Algeria

^7^ Department of Drugs Industry and Pharmaceutical Management, George Emil Palade University of Medicine, Pharmacy, Science and Technology of Târgu Mures, Romania

^8^ Sz-imfidum Ltd., 525401 Lunga, Romania

* Corresponding author: Gergő Tóth, Department of Pharmaceutical Chemistry, Semmelweis University, H-1092 Budapest, Hőgyes E. u. 9, Hungary.

Tel: +36 12170891; Fax: +36 12170891

Email address: toth.gergo@semmelweis.hu

**Supplementary Table 1** Chromatographic data on Cyclobond I 2000 column (*k*_1_ – retention factor of the first eluting enantiomer, *k*_2_ - retention factor of the second eluting enantiomer, α – selectivity, *R*_s_ – resolution)

|  |  |  | MeOH* | | |  | ACN* | | | |
| --- | --- | --- | --- | --- | --- | --- | --- | --- | --- | --- |
|  |  | *k*_1_ | *k*_2_ | *α* | *R*_s_ | *k*_1_ | | *k*_2_ | *α* | *R*_s_ |
|  | Stiripentol | 0.10 | - | - | - | 0.03 | | - | - | - |
|  | Thalidomide | 0.16 | - | - | - | 0.02 | | - | - | - |
|  | Pomalidomide | 0.19 | - | - | - | 0.06 | | - | - | - |
|  | Lenalidomide | 0.20 | - | - | - | 0.28 | | - | - | - |
|  | Apremilast | 0.20 | - | - | - | 0.01 | | - | - | - |
|  | Neutral iminoflavan | 0.12 | - | - | - | 0.02 | | - | - | - |
|  | Guaifenesin | 0.09 | - | - | - | 0.32 | | - | - | - |
|  | Naproxen | 2.51 | - | - | - | 0.67 | | - | - | - |
|  | Ibuprofen | 1.89 | - | - | - | 0.61 | | - | - | - |
|  | Warfarin | 0.12 | - | - | - | 1.01 | | 1.11 | 1.10 | 1.2 |
|  | Naringenin | 0.16 | - | - | - | 1.00 | |  |  |  |
|  | Hesperetin | 0.18 | - | - | - | 0.66 | |  |  |  |
|  | Bamethan | 0.11 | - | - | - | 0.08 | |  |  |  |
|  | Terbutaline | 0.13 | - | - | - | 0.08 | |  |  |  |
|  | Amphetamine | 0.28 | - | - | - | 0.08 | |  |  |  |
|  | Methamphetamine | 0 | - | - | - | 0.24 | |  |  |  |
|  | 1-aminoindane | 0.09 | - | - | - | 0.03 | |  |  |  |
|  | Propranolol | 0.15 | - | - | - | 2.96 | |  |  |  |
|  | Metoprolol | 0.14 | - | - | - | 2.90 | | 3.20 | 1.11 | 0.9 |
|  | Bisoprolol | 0.10 | - | - | - | 3.53 | |  |  |  |
|  | Basic Iminoflavan | 0.12 | - | - | - | 0.10 | |  |  |  |
|  | Ofloxacin | 0.78 | - | - | - | 1.39 | |  |  |  |
|  | Norepinephrine | 0.59 | - | - | - | 0.89 | |  |  |  |
|  | Omeprazole | 0.17 | - | - | - | 1.22 | | 1.24 | 1.01 | 0.3 |
|  | Rabeprazole | 0.16 | - | - | - | 1.45 | | 1.49 | 1.02 | 0.5 |

* For neutral compounds (**1-7**) pure eluent, for acidic compounds (**8-12**) the eluent was modified with 0.1% Acac (v/v), for basic compounds (**13-21**) the eluent was modified with 0.1% DEA (v/v), for amphoteric compounds (**22-25**) the eluent was modified with the mixture of DEA-Acac 0.1%-0.1% (v/v).

**Supplementary Table 2** Chromatographic data on Nucleodex β-PM column (*k*_1_ – retention factor of the first eluting enantiomer, *k*_2_ - retention factor of the second eluting enantiomer, α – selectivity, *R*_s_ – resolution)

|  |  |  | MeOH* | | |  | ACN* | | | |
| --- | --- | --- | --- | --- | --- | --- | --- | --- | --- | --- |
|  |  | *k*_1_ | *k*_2_ | *α* | *R*_s_ | *k*_1_ | | *k*_2_ | *α* | *R*_s_ |
|  | Stiripentol | 0.26 | - | - | - | 0.04 | | - | - | - |
|  | Thalidomide | 0.43 | - | - | - | 0.01 | | - | - | - |
|  | Pomalidomide | 0.54 | - | - | - | 0.06 | | - | - | - |
|  | Lenalidomide | 3.42 | - | - | - | 0.14 | | - | - | - |
|  | Apremilast | 0.39 | - | - | - | 0.01 | | - | - | - |
|  | Neutral iminoflavan | 0.71 | - | - | - | 0.03 | | - | - | - |
|  | Guaifenesin | 0.08 | - | - | - | 0.21 | | - | - | - |
|  | Naproxen | 0.33 | - | - | - | 0.29 | | - | - | - |
|  | Ibuprofen | 0.18 | - | - | - | 0.26 | | 0.28 | 1.08 | 0.4 |
|  | Warfarin | 0.24 | - | - | - | 0.02 | | - | - | - |
|  | Naringenin | 0.70 | - | - | - | 0.83 | | - | - | - |
|  | Hesperetin | 0.77 | 0.82 | 1.06 | 0.3 | 0.41 | | 0.55 | 1.35 | 0.8 |
|  | Bamethan | 0.24 | - | - | - | 0.03 | |  |  |  |
|  | Terbutaline | 0.26 | - | - | - | 0.03 | |  |  |  |
|  | Amphetamine | 0.64 | - | - | - | 0.03 | |  |  |  |
|  | Methamphetamine | 0.56 | - | - | - | 0.03 | |  |  |  |
|  | 1-aminoindane | 0.22 | - | - | - | 0.06 | |  |  |  |
|  | Propranolol | 0.38 | - | - | - | 0.05 | |  |  |  |
|  | Metoprolol | 0.23 | - | - | - | 0.03 | | - |  |  |
|  | Bisoprolol | 0.14 | - | - | - | 0.03 | |  |  |  |
|  | Basic Iminoflavan | 0.58 | 0.62 | 1.07 | 0.5 | 0.05 | |  |  |  |
|  | Ofloxacin | 0.06 | - | - | - | 0.98 | |  |  |  |
|  | Norepinephrine | 0.13 | - | - | - | 0.80 | |  |  |  |
|  | Omeprazole | 0.14 | - | - | - | 0.26 | |  |  |  |
|  | Rabeprazole | 0.10 | - | - | - | 0.25 | | 0.36 | 1.43 | 0.5 |

* For neutral compounds (**1-7**) pure eluent, for acidic compounds (**8-12**) the eluent was modified with 0.1% Acac (v/v), for basic compounds (**13-21**) the eluent was modified with 0.1% DEA (v/v), for amphoteric compounds (**22-25**) the eluent was modified with the mixture of DEA-Acac 0.1%-0.1% (v/v).

**Supplementary Table 3** Chromatographic data on Lux amylose-1 column (*k*_1_ – retention factor of the first eluting enantiomer, *k*_2_ - retention factor of the second eluting enantiomer, α – selectivity, *R*_s_ – resolution)

|  |  |  | MeOH* | | |  | ACN* | | | |
| --- | --- | --- | --- | --- | --- | --- | --- | --- | --- | --- |
|  |  | *k*_1_ | *k*_2_ | *α* | *R*_s_ | *k*_1_ | | *k*_2_ | *α* | *R*_s_ |
|  | Stiripentol | 0.87 | 1.42 | 1.64 | 5.4 | 0.75 | | 1.46 | 1.96 | 4.9 |
|  | Thalidomide | 3.30 | 5.25 | 1.58 | 7.7 | 1.89 | | 1.98 | 1.05 | 0.3 |
|  | Pomalidomide | 3.77 | 6.43 | 1.70 | 8.1 | 3.47 | | - | - | - |
|  | Lenalidomide | 0.67 | 1.24 | 1.84 | 3.5 | 1.23 | | 1.48 | 1.20 | 1.5 |
|  | Apremilast | 2.87 | - | - | - | 0.44 | | 0.73 | 1.67 | 1.6 |
|  | Neutral iminoflavan | 3.01 | 4.17 | 1.39 | 5.7 | 0.66 | | 1.33 | 2.00 | 6.5 |
|  | Guaifenesin | 0.25 | 0.31 | 1.23 | 1.2 | 0.42 | | 0.51 | 1.21 | 1.1 |
|  | Naproxen | 0.52 | 0.56 | 1.08 | 0.4 | 0.53 | | 0.60 | 1.12 | 0.7 |
|  | Ibuprofen | 0.19 | 0.21 | 1.11 | 0.3 | 0.47 | | - | - | - |
|  | Warfarin | 0.60 | 1.32 | 2.16 | 1.7 | 0.35 | | 0.61 | 1.73 | 1.7 |
|  | Naringenin | 2.24 | 2.98 | 1.33 | 2.32 | 0.52 | | - | - | - |
|  | Hesperetin | 4.54 | 4.88 | 1.07 | 0.8 | 1.19 | | 1.49 | 1.26 | 0.7 |
|  | Bamethan | 0.10 | - | - | - | 0.80 | |  |  |  |
|  | Terbutaline | 0.05 | - | - | - | 0.89 | |  |  |  |
|  | Amphetamine | 0.35 | 0.39 | 1.13 | 0.3 | 1.05 | | 1.30 | 1.24 | 1.9 |
|  | Methamphetamine | 0.36 | - | - | - | 1.12 | | 1.33 | 1.18 | 2.2 |
|  | 1-aminoindane | 0.89 | 1.04 | 1.17 | 0.8 | 1.20 | | 1.41 | 1.18 | 1.94 |
|  | Propranolol | 0.39 | 0.81 | 2.07 | 3.5 | 1.15 | |  |  |  |
|  | Metoprolol | 0.84 | 1.15 | 1.36 | 2.7 | 3.93 | | - |  |  |
|  | Bisoprolol | 0.61 | 0.72 | 1.17 | 1.1 | 3.08 | |  |  |  |
|  | Basic Iminoflavan | 2.87 | 3.81 | 1.32 | 4.54 | 0.57 | | 1.16 | 2.03 | 5.7 |
|  | Ofloxacin | 3.30 | - | - | - | 1.41 | | 1.49 | 1.06 | 0.3 |
|  | Norepinephrine | 2.47 | 2.62 | 1.06 | 0.8 | 4.77 | |  |  |  |
|  | Omeprazole | 2.85 | 3.48 | 1.22 | 1.3 | 7.96 | | 10.94 | 1.37 | 1.6 |
|  | Rabeprazole | 2.03 | 2.29 | 1.12 | 1.5 | 5.91 | | - | - | - |

* For neutral compounds (**1-7**) pure eluent, for acidic compounds (**8-12**) the eluent was modified with 0.1% Acac (v/v), for basic compounds (**13-21**) the eluent was modified with 0.1% DEA (v/v), for amphoteric compounds (**22-25**) the eluent was modified with the mixture of DEA-Acac 0.1%-0.1% (v/v).

**Supplementary Figure 1** Characterization of the success of the applied separation system. Separation conditions were as indicated in “Materials and methods” section. (The success of the separation system was evaluated using three approaches: the ∑Rs approach, the scoring approach, and the 0-1 approach. For the ∑Rs approach, the sums of the individual Rs values were calculated. In the scoring approach, a scoring system was applied: 0 if Rs=0, 1 if Rs<1.5, 2 if 1.5<Rs<2.5, 3 if 2.5<Rs<4, and 4 if Rs>4. For the 0-1 approach, a value of 1 was assigned if enantiorecognition was observed and 0 if not.)

**Supplementary Table 4** Resolution values (*R*_s_) for basic compounds at different DEA percentages in MeOH

| **Compounds** | neat MeOH | MeOH +0.05% DEA | MeOH +0.1% DEA | MeOH +0.15% DEA |
| --- | --- | --- | --- | --- |
| Bisoprolol | No separation | 2.00 | 2.10 | 2.23 |
| Metoprolol | No separation | 1.41 | 1.52 | 1.77 |
| Basic Iminoflavan | 4.62 | 4.61 | 4.64 | 4.57 |

**Supplementary Table 5** Resolution values (*R*_s_) for acidic compounds at different acetic acid (Acac) percentages in MeOH

| **Compounds** | neat MeOH | MeOH +0.05% Acac | MeOH +0.1% Acac | MeOH +0.15% Acac |
| --- | --- | --- | --- | --- |
| Hesperetin | 2.75 | 2.83 | 3.0 | 2.89 |
| Naringenin | 0.51 | 0.54 | 0.62 | 0.58 |

**Supplementary Table 6** Resolution values (*R*_s_) for amphoteric compounds at different acetic acid (Acac) and DEA percentages in MeOH

|  | Neat MeOH | MeOH  +0.05%DEA | MeOH  +0.1%DEA | MeOH  +0.15%DEA | MeOH  +0.05% Acac | MeOH  +0.1% Acac | MeOH  +0.15% Acac |
| --- | --- | --- | --- | --- | --- | --- | --- |
| Norepinephrine | No separation | 1.48 | 1.47 | 1.42 | No separation | No separation | No separation |
| Rabeprazole | 1.20 | 0.51 | No separation | No separation | No separation | No separation | No separation |

|  | MeOH  +0.05%DEA +0.05% Acac | MeOH  +0.1%DEA + 0.1% Acac | MeOH  +0.15% DEA + 0.15 Acac |
| --- | --- | --- | --- |
| Norepinephrine | 1.43 | 1.54 | 1.40 |
| Rabeprazole | 1.39 | 1.48 | 1.29 |


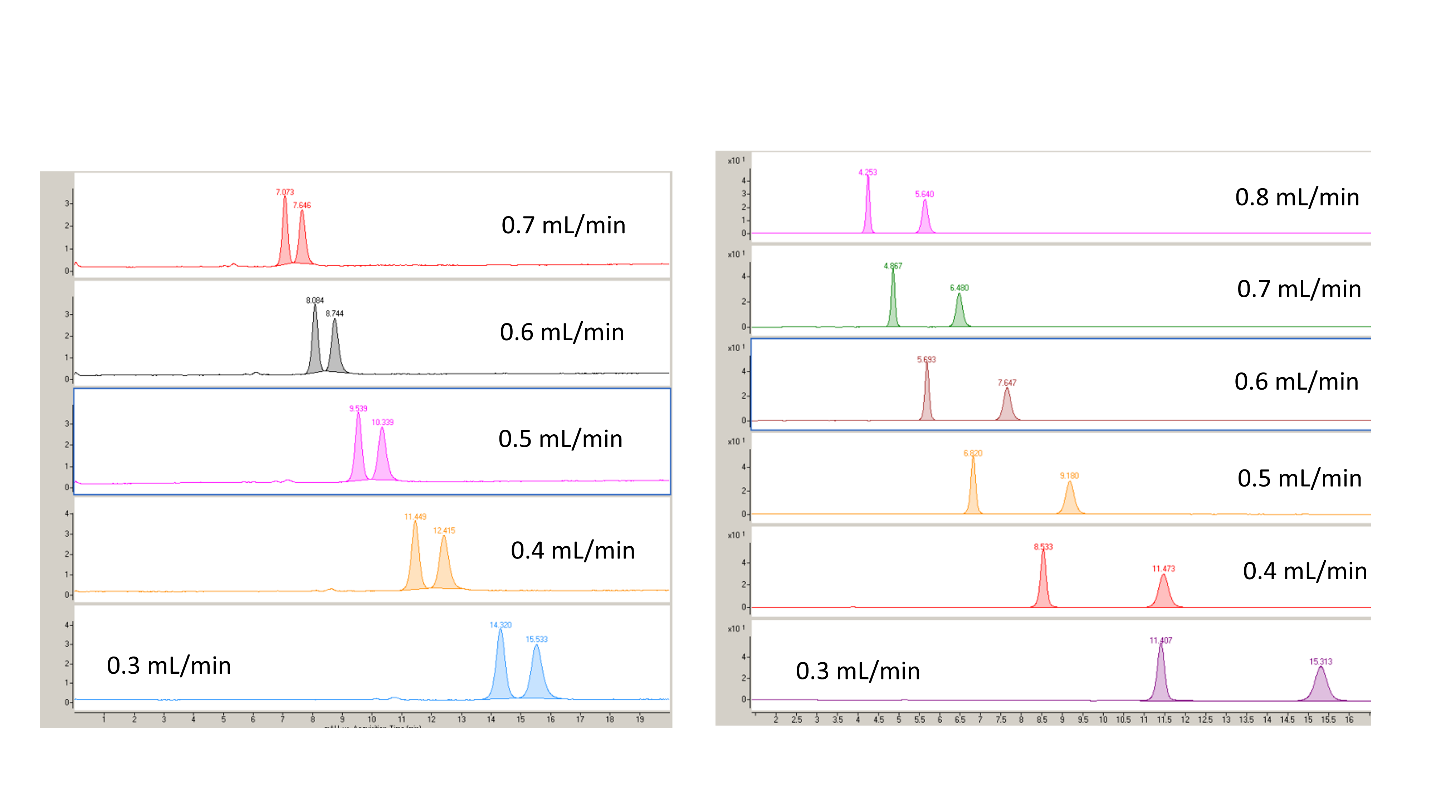


**Supplementary Figure 2** Separation of lenalidomide (left) and stiripentol (right) using different flow rate in neat methanol at 25 °C.


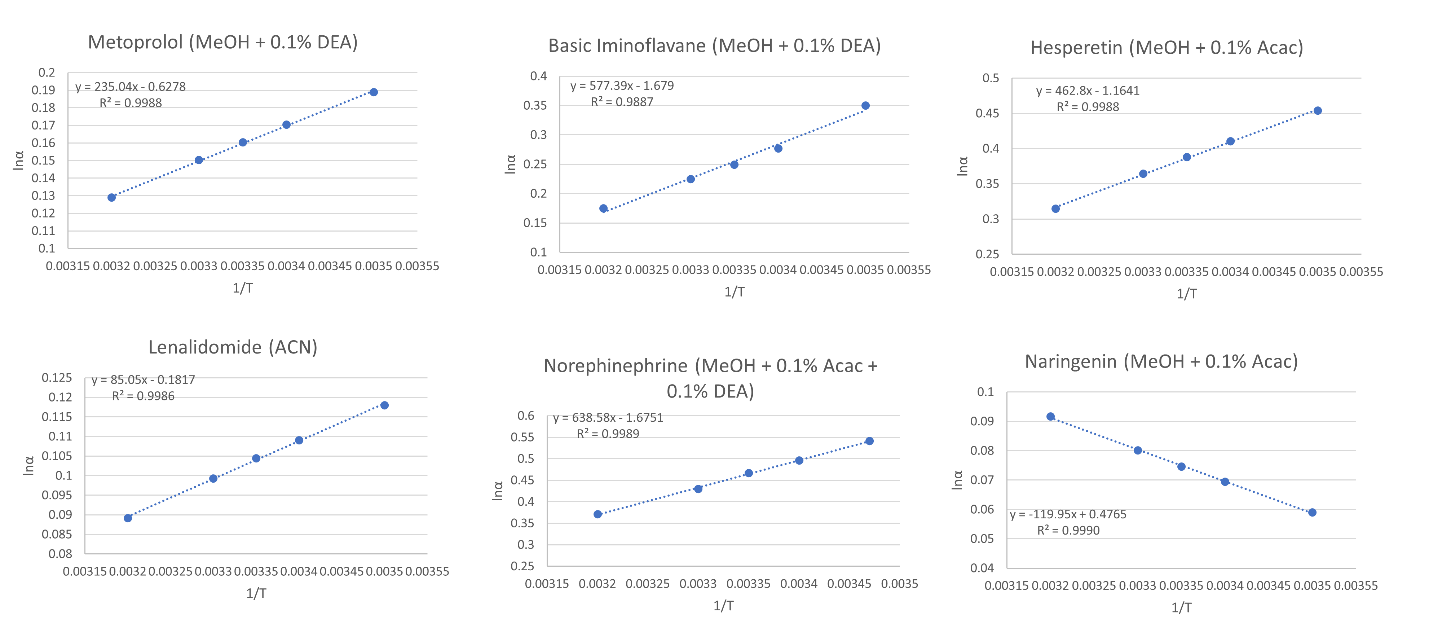


**Supplementary Figure 3** lnα vs. 1/T curves for different analytes on Chiral CD-Ph column. Flow rate: 0.5 mL/min. Temperature range 10-40 °C.

*
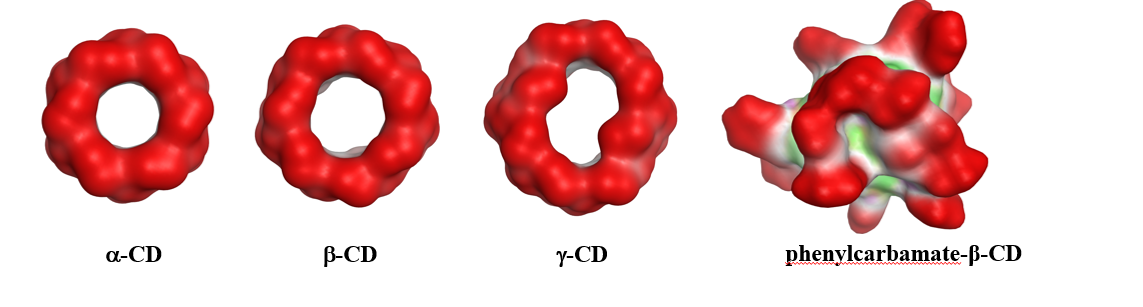
*

**Supplementary Figure 4** The molecular surface of the 3D structure of α-CD, β-CD, γ-CD, and phenylcarbamate-β-CD, respectively.

**Supplementary Table 7** Comparison of the results of docking and single-point energy calculations

|  | **Docking** | **APFD/6-31G(d)** |
| --- | --- | --- |
|  | ***ΔE*__R-S_** | |
|  | **kcal/mol** | |
| **Thalidomide** | -0.5 | -2.9 |
| **Pomalidomide** | -0.5 | -1.4 |
| **Stiripentol** | -0.1 | -4.8 |
